# Interpreting Mamelodi Community-Oriented Primary Care data on tuberculosis loss to follow-up through the lens of intersectionality

**DOI:** 10.4102/phcfm.v12i1.2081

**Published:** 2020-02-04

**Authors:** Blandine B. Ilunga, Owen O. Eales, Tessa S. Marcus, Selma Smith, Jannie F. Hugo

**Affiliations:** 1 Department of Family Medicine, School of Medicine, Faculty of Health Sciences, University of Pretoria, Tshwane, South Africa; 2 Kalafong Provincial Tertiary Hospital, Tshwane, South Africa

**Keywords:** Loss to Follow-Up, Tuberculosis, Social Determinants, Intersectionality, Community Health Workers, Community-Oriented Primary Care, eHealth

## Abstract

**Background:**

Tuberculosis (TB) is a persistent major public health challenge in South Africa. This article examines the social determinants and demographic factors associated with TB loss to follow-up through the lens of intersectionality.

**Aim:**

The aim of this study was to describe and interpret the social determinants and demographic factors associated with TB patients lost to follow-up (LTFU).

**Setting:**

Mamelodi, an urban settlement in the South African District of Tshwane.

**Methods:**

AitaHealth™ is an Information and Communications Technology (ICT) mobile and web application that is used by community health workers. Data from patients with TB were extracted from the 64 319 households registered on AitaHealth™ over a 3-year period. Univariate and multivariate analyses were used to compare patients who were adherent to TB treatment and those LTFU.

**Results:**

Of the 184 351 individuals screened for TB, 788 reported that they were diagnosed with TB (an incidence of 427 cases per 100 000). Of the 704 eligible for inclusion in this analysis, 540 (77%) were on treatment and 164 (23%) were LTFU. The factors associated with LTFU were aged over 60, not having a South African identification document, migration and death in the household, and higher mean household income.

**Conclusion:**

The results of this study serve as a reminder to clinicians of the importance of the three-stage assessment (biopsychosocial) in the approach to patients with TB. Understanding the intersection of social determinants and demographic factors helps clinicians and others identify and respond to the specificity of patient, health system and non-health policy issues at play in LTFU.

## Introduction

R. was a gardener and worked at two locations 6–7 days a week. He came from Malawi and had been living in South Africa for the last 7 years. He supported his girlfriend and also sent money to his children in Malawi.

In August 2018, he started coughing, losing weight and feeling weak. Apart from just hoping that he would soon get better, he did not seek medical help for a number of reasons. Firstly, he did not want to waste days of pay waiting all day at the clinic. Secondly, he did not feel that he could afford the expense of seeing a doctor, and finally he was afraid of losing his job and thus hid his medical problems from his employers.

Too weak to work, he finally went to the clinic where he was diagnosed with Tuberculosis (TB) and Human Immunodeficiency Virus (HIV). He did not accept the diagnosis. He believed that his symptoms were caused by bewitchment. After counselling at the local clinic as well as with extensive support and counselling from one of his employers, he agreed to take his medication. Because he had delayed treatment, his condition did not improve immediately. This fact fuelled his suspicion of bewitchment.

He worried about his livelihood. One employer stopped paying him. The other continued to support him without the expectation of him working. And because he was a foreign national, he did not have access to the temporary disability grant for severe TB that is afforded to South Africans.

He stopped taking his medication regularly and consulted with a traditional healer. His condition deteriorated, he struggled to walk and became disoriented. He died in January 2019.

South Africa has the world’s sixth largest TB epidemic,^1^ with approximately 1% of the population developing active TB every year.^2^ It also has 7.2 million people living with HIV (2017) and an HIV prevalence of 18.9% (among people aged 15–49 years).^3^

Globally, TB has significantly complicated the HIV epidemic over the past two decades.^4^ It accounted for about one in four deaths of HIV-infected patients worldwide^5^ and more than half the deaths of people with HIV in South Africa, where an estimated 60% of HIV+ people have TB. Being HIV+ increases the risk of developing active TB 20- to 30-fold.^6^

Tuberculosis is a persistent and difficult condition to contain. The activation of TB and individual, institutional and system responses to it are the result of a complex interface of biological, social and environmental factors. These are structural, physical and social at the same time as they are embodied in individual and collective agency.

Existing understanding of the infectious agent *Mycobacterium tuberculosis* and the disease course it triggers has led to the development of effective prevention and treatment regimens even in a context of HIV and drug-resistant TB. Early treatment and treatment adherence lead to the restoration of patient health and at the same time helps control the spread of the disease.^7^ Isoniazid Preventive Therapy (IPT) significantly reduces the risk of TB activation in people infected with HIV and treatment.^8^ Testing helps prevent infection in families, communities and healthcare providers exposed to active TB. Conversely, treatment delay or interruption is a significant contributor to mortality and morbidity among TB patients and is an important cause of drug-resistant TB.^9^ In South Africa, nearly one-third of all diagnosed TB patients (32.8%) are lost to follow-up (LTFU) in the existing facility-based healthcare system. Most are initial LTFU (27.2%), where patients are diagnosed with TB but receive no treatment.^2,10^ The remaining 5.6% of all TB diagnosed patients LTFU is where patients have interrupted treatment for 2 or more months.^2,11^ Loss to follow-up outside of the clinical setting as contact tracing, screening and IPT initiation is often poorly implemented for adults, and especially for children, in South Africa and elsewhere.^8^

At a macro-level, TB transmission and treatment initiation, adherence and outcomes are generally associated with class, colour, origin, age and sex, as they manifest in income, education, occupation, shelter, food security, gender and marital status, migration and so on.^12,13,14,15,16^

However, it is difficult to translate these structural ‘cause of causes’ practically, either in the course of clinical care at an individual level or organisationally through the institutions of service delivery. There are several reasons for this. Social determinants fail to account for agency or the way people, individually and through institutions, act on the world even when they are constrained by the place, time and circumstances of their birth.^17^ Social structural causes fail to engage with the institutional arrangements that organise the distribution of services, resources and power. Conceptually, they are often construed as being hierarchically ordered in historically predetermined ways.^18^ And practically, they operate at a level of abstraction that is far removed from their embodiment in individuals and interpersonal relations. Simply put, they are difficult to distil to an out-of-context individual in a clinical encounter.

Gaps in knowledge and system responses evidenced by the failure to effectively contain the TB epidemic have seen some important paradigmatic and conceptual shifts. Significant basic science breakthroughs in HIV prevention and treatment have driven a paradigm shift in biophysical TB research. The global effort is now focused on the elucidation of biomarkers to ‘accurately and promptly predict key active or latent TB. In much the same way that the identification of HIV viral load accurately predicts the development of opportunistic infections and mortality.’^19^

Similarly, there have been shifts in society and system paradigms. Specifically, the notions of complex adaptive systems and, within them, intersectionality provide a way of accounting for the complexity of structure, system and agency in health and healthcare.^18^ Complex adaptive systems are made up of many parts that act on and react to each other in dynamic and evolutionary, non-linear ways. Intersectionality helps us consider simultaneity as it occurs in individuals and in the relations between individuals, or the co-occurrence and interaction of social determinants and systems that inform agency.

Institutionally, there is increasing awareness globally about the need to reconceptualise the healthcare system in a way that prioritises primary care and extends healthcare services to and from people in their homes and communities to achieve health for all.^20^ This is presently articulated in South Africa through the envisaged creation of a national health insurance to drive sector integration and cost-effective, quality service delivery.^21^ Under the policy umbrella of primary care re-engineering, it has been practically developed and applied as 21st-century electronic Information and Communications Technology (ICT)-enabled Community-Oriented Primary Care (COPC). Information and Communications Technology-enabled COPC mobilises clinical services and public health resources to deliver healthcare in the places where people live and work. It is geographically focused, person- and family-centred, comprehensive, generalist and equitable healthcare. It is delivered by multi-disciplinary teams, and informed by the best available practices and knowledge. It uses AitaHealth™, a web and mobile electronic platform that records, guides and reports healthcare information and services provided by health workers to individuals and families in their homes and communities. Purposively designed by the University of Pretoria and Mezzanineware/Vodacom to enable real-time communication and data-informed decision support and service management, it is used by Ward-Based Outreach Teams (WBOTs) to register, assess and triage households and individuals according to national health disease and condition priorities, including TB.^22^

## Method

### Study design

A cross-sectional, descriptive analysis of demographic and social factors associated with TB treatment LTFU.

### Setting

Mamelodi (Gauteng, South Africa) is a 45.2 km^2^ urban settlement in the east of the City of Tshwane. It has a population of 334 577 people living in approximately 110 703 households. Nearly all residents (98.8%) are African and 42.5% speak Sepedi as the first language. One-third of the households are female headed and 40% live on less than R20 000 a year. The majority (61%) of people reside in formal dwellings. About two-fifths (38.4%) of the population above the age of 20 years have completed secondary school.^23^

### Selection and data collection

The study population comprised 64 319 registered households, with 184 354 people who were screened for TB over the 3-year period, 01 August 2014–10 October 2017 (> 50% of the total Mamelodi population). All individuals, 18 years or older, captured on AitaHealth™ by community healthcare workers (CHWs), who were diagnosed with TB, were included in the study. Those who did not give consent for their data to be used for research purposes were excluded.

### Data analysis

The following demographic and social factors were analysed, namely, age, sex, relationship status, possession of a South African Identification Document (ID), in- and out-migration, food security status, type of dwelling, ownership of the dwelling, death in the household and household income.

Data were cleaned and entries with contradictory information regarding treatment status were excluded. Cleaned data were then grouped into two, namely, TB treatment adherent and LTFU. The data were analysed using EpiData software. Groups were compared using Fisher’s exact test at the 0.05 level of significance. Odds Ratios (ORs) for all factors were reported along with a 95% Confidence Interval (CI). Informed consent was obtained on AitaHealth™ for both service and research.

### Ethical considerations

Ethical approval for the study was obtained from the Faculty of Health Sciences Research Ethics Committee of the University of Pretoria (Ethics reference number: 286/2018).

## Results

Of the 184 351 individuals screened for TB, 788 reported that they were diagnosed with TB. This equates to an incidence of 427 cases per 100 000 population over the 3-year period. Respondents below 18 years of age (84) were excluded. Of the 704 eligible for inclusion in this analysis, 540 (77%) were on treatment and 164 (23%) were LTFU.

Most (58%) of the respondents were male, single (68%) and of formal working age (18–59) (89%). Sixty-one per cent were foreigners and did not have a South African ID document (Table 1).

**TABLE 1:** Socio-demographic characteristics of people with tuberculosis.

Characteristics	Number of participants *n* = 704	
	*N*	%
**Age (*n* = 693)**		
18–40 years	326	47
>40–60 years	297	43
>60 years	70	10
**Sex (*n* = 704)**		
Male	408	58
Female	296	42
**Possession of RSA ID (*n* = 704)**		
Possess	272	39
Does not possess	432	61
**In-migration in last 12 months (*n* = 615)**		
No	592	96
Yes	23	4
**Out-migration in last 12 months (*n* = 691)**		
No	629	91
Yes	62	9
**Food security (*n* = 628)**		
Food security	527	84
Food insecurity	101	16
**Dwelling type (*n* = 621)**		
House	365	59
Shack	208	33
Room	48	8
**Ownership of dwelling (*n* = 690)**		
Own the dwelling	573	83
Do not own the dwelling	117	17
**Death in the household (*n* = 691)**		
No death within 12 months	599	87
Death within 12 months	92	13
**Groups (*N* = 704)**		
Adherent to treatment	540	77
LTFU	164	23

ID, identification document; LTFU, lost to follow-up.

Most (83%) of the respondents lived in dwellings owned by them or their families, although only 59% lived in formal houses. While few households (*n* = 23, 4%) had experienced in-migration of a household member, nearly three times as many (*n* = 62, 9%) had experienced out-migration in the previous 12 months. Sixteen per cent were food insecure and 13% had experienced a death in the household within the previous 12 months. The median household income was R2000 per month in the 161 (23%) individuals who reported on this item.

Univariate and multivariate comparison of the socio-demographic characteristics of individuals who are TB treatment adherent and those LTFU found several important differences (see Table 1).

People who did not possess a South African ID were twice as likely to be LTFU compared with those who possessed. Also, individuals with TB were three times more likely to be LTFU where there had been a death in the household (Table 2).

**TABLE 2:** A comparison of selected socio-demographic characteristics of people with tuberculosis in treatment and lost to follow-up.

Exposure variables	In treatment		LTFU		Univariate analysis		Multivariate analysis	
	*n*	%	*n*	%	Crude OR	*p*	Adjusted OR	*p*
**Age (*n* = 693)**								
18–40 years	243/532	46.0	83/161	52.0	1.00	-	1.00	-
> 40–60 years	245/532	46.0	52/161	32.0	0.62	0.017	0.75	0.220
> 60 years	44/532	8.0	26/161	16.0	1.70	0.045	2.04	0.031
**Possession of South African ID (*n* = 704)**								
Possess	229/540	42.4	43/164	26.2	1.00	-	1.00	-
Not possess	311/540	57.6	121/164	73.8	2.07	< 0.001	2.30	0.021
**In-migration within last 12 months (*n* = 616)**								
No	476/485	98.1	116/130	89.2	1.00	-	1.00	-
Yes	9/485	1.9	14/130	10.8	6.38	< 0.001	3.12	0.022
**Out-migration within last 12 months (*n* = 691)**								
No	506/535	94.6	123/156	78.8	1.00	-	1.00	-
Yes	29/535	5.4	33/156	21.2	4.68	< 0.001	2.75	0.006
**Household deaths within the past 12 months (*n* = 691)**								
No death	485/535	90.6	114/156	73.1	1.00	-	1.00	-
Death	50/535	9.4	42/156	26.9	3.70	< 0.001	1.83	0.058

Note: Bold values indicate *p* < 0.05 as significant or between 0.05 and 0.01 as marginally significant.

OR, odds ratio; ID, identification document; LTFU, lost to follow-up.

Comparing income data, available for 32% (53/164) of the LTFU group and 20% (109/540) of the treatment adherent group, the median income of those who were LTFU (R3000) was 50% more than those who adhered to TB treatment (R2000).

In multivariate analysis, not possessing a South African ID (*p* = 0.015), a death in the household (*p* = 0.015) and out-migration, where a household member left the household in the previous 12 months (*p* = 0.012), were found to be significantly associated with LTFU.

## Discussion

This study found a TB incidence of 427 per 100 000 over the 3-year period, which is lower than the 2017 WHO South Africa TB estimate of 567 per 100 000^24^ (WHO TB burden estimates). Although not strictly comparable in terms of timeframe, it is likely that the prime reason for the difference in incidence in this geographic community compared to the country as a whole is that the total population data conceal local variability and the importance of place. It is well known that the incidence of many conditions varies by locality. For example, even in a generalised HIV epidemic where TB is the primary opportunist infection, the TB incidence in Southern Africa is higher in mining and mining labour-supply communities compared to those where people are not involved in mineral extraction.^25^

What these locality-specific data collected by CHWs doing IT-enabled COPC provide is an understanding of micro-level incidence. Not only is such information generally not available, it is also not usually linked in real time to a healthcare service that extends delivery to and from homes. Using AitaHealth™, healthcare professionals and workers involved in community-based and facility primary healthcare are able to access person- and patient-specific data to inform prevention and treatment practice in context.

The definition of LTFU with respect to TB is either being diagnosed but not starting treatment (also called initial LTFU) or once initiated, interrupting treatment for 2 or more consecutive months (formerly termed treatment defaulters).^11^ In this study, nearly a quarter (23%) of TB diagnosed patients were LTFU, which is high, even though it is lower than the national estimate of 32.8%. The official percentage of LTFU is 5.6%, but if we include the initial LTFU at 27.2%,^10^ the percentage increases to 32.8% or nearly one-third of all diagnosed TB patients.

Similar to international research, the results of this study show that age, particularly being older (> 60 years), is associated with LTFU. However, no association was found between sex and TB LTFU, contrary to other evidence of clear significant associations between being male and defaulting TB treatment.^26,27^

Two of the commonly known socio-economic drivers of TB LTFU were found in this study. Loss to follow-up was significantly associated with the migration of a household member out of the area within the previous 12 months as was being foreign or without having a South African ID. The pull and push for internal and international migration is most often related to the pursuit of economic opportunities and/or the escape of war, conflict and political as well as domestic repression. Inevitably migration also results in the disruption and loss of structural and emotional support systems and at the same time as it engenders the necessity of negotiating unfamiliar, sometimes hostile cultural and institutional environments.^28^ There is strong evidence that people without South African IDs and internal migrants living in urban peripheries face documentation, language and service access barriers to healthcare^29^ as well as discriminatory and disrespectful in-facility care.^30^

The finding that death in the household in the preceding 12 months is marginally associated with LTFU (*p* = 0.058) speaks about two dimensions of the challenge of treating TB, namely, one relates to the importance of the family in treatment adherence,^31^ especially the role of lack of social support in negative treatment outcomes,^32^ and the other relates to the infectious nature of TB that puts all household members at risk of infection and death. Debilitated by TB, the association of death and TB in a household impacts people’s sense of self-worth and self-efficacy, as well as their interpersonal relations, especially the ability and desire of others to support them through their illness, including starting and staying on treatment.^33^

Contrary to expectation, the LTFU group had a higher mean household income (R3000) than those in the adherent group (R2000). It would seem that in a context of relative poverty, a risk to LTFU is the struggle to keep income and resist descent into greater poverty. Although this finding needs to be treated with some caution as it is based on only a subset of respondents, it is consistent with other studies that have shown that TB worsens poverty because of loss of income incurred in seeking healthcare and that this loss is greatest in the period prior to starting treatment.^34^

While each of these socio-economic and demographic factors provides insights into the structural determinants that underlie LTFU, it is in their intersection that their impact becomes significant. Intersectionality helps frame the relationship between factors in a way that is meaningful and practical. In this specific context, LTFU was found at the intersection of being physically, socially and institutionally ‘out of place’ (migrant, foreign) and a part of the relatively better-off poor. Intersectionality also talks about the fact that determinants are embodied in individuals and in the power relationships between people. They inform agency or what people think and feel they can do about conditions and circumstances, be they patients, healthcare practitioners, family, friends and community members. This makes the concept of intersectionality practically useful because it links the understanding of health determinants to the complexity of people’s responses at the micro-level as well as to their relationships at meso- and macro-levels.

## Conclusion

The coalface of healthcare, including TB LTFU and other impediments to treatment, is constituted and reconstituted by the intersections of multi-dimensional, socially embodied people in relationship to themselves and others in time and place.

For clinicians practising patient-centred care, this study shows that understanding intersectionality is key to doing effective three-stage (biopsychosocial) assessments^35^ and developing mutually agreed plans that actually mobilise people’s ability to make choices.

For healthcare service managers, the study shows that health responses and outcomes are shaped by the intersection of systems and people in their homes and communities. To get ahead of the TB epidemic, it is essential to create a coordinated comprehensive system that could provide services to people to and from the home and facilities in ways that support equity.

For policymakers, the study shows how societal and policy issues that extend beyond the direct remit of health systems directly and negatively impact efforts to contain TB. Health-for-all means ensuring that all sectors and services are aligned to protect the vulnerable and the weak.

## Strengths and limitations

### Strengths

The data were captured electronically and referenced to a GPS coordinate. The data were captured over a 3-year period and thus were not influenced by seasonal fluctuations in LTFU. The data were collected at household level by CHWs. This study included all initial LTFU data (patients diagnosed with TB but not initiated on TB treatment).

### Limitations

The questions that informed LTFU in this study are not exactly the same as the official WHO definition. We asked about household members who have been diagnosed with TB in the last 12 months and have defaulted or stopped TB treatment. The 2013 WHO definition of LTFU is as follows, namely, a TB patient who has interrupted or stopped TB treatment for more than 2 months. This definitional difference may account for some of the variations between site-specific and national incidence. The data were captured by CHWs with different experience and training, which could influence data quality. The data only show an association with LTFU and causality cannot be inferred. Household data used in this study are self-reported and may be influenced by difficulty in recall and response bias.^36^
